# Treatment options in hypoparathyroidism

**DOI:** 10.20945/2359-3997000000554

**Published:** 2022-11-10

**Authors:** Eliane Naomi Sakane, Maria Carolina Camargo Vieira, Gabriela Mazzarolo Marcondes Vieira, Sergio Setsuo Maeda

**Affiliations:** 1 Universidade Federal de São Paulo Disciplina de Endocrinologia Departamento de Medicina São Paulo SP Brasil Departamento de Medicina, Disciplina de Endocrinologia, Universidade Federal de São Paulo (Unifesp), São Paulo, SP, Brasil

**Keywords:** Hypoparathyroidism, treatment, PTH analogs, vitamin D, perspectives

## Abstract

Hypoparathyroidism remains the single endocrine deficiency disease that is not habitually treated with the missing hormone. In this article, we aim to provide a review of the conventional approach and the novel therapies as well as an overview of the perspectives on the treatment of this rare condition. We conducted a literature review on the conventional therapy using vitamin D analogs and calcium salts, indications for thiazide diuretics and phosphorus binders, PTH analogs history and usage, and the drugs that are currently being tested in clinical trials. Conventional treatment involves calcium salts and vitamin D analogs. Thiazide diuretics can be used to reduce hypercalciuria in some cases. A low-phosphate diet is recommended, and phosphate binders are rarely needed. During pregnancy, a careful approach is necessary. The use of PTH analogs is a new approach despite the limitation of high cost. Studies have included modified PTH molecules, calcilytics, microencapsulation of human parathyroid cells, and allotransplantation.

## INTRODUCTION

Hypoparathyroidism is a rare endocrine disease characterized by hypocalcemia associated with hyperphosphatemia and concomitant absent or inappropriately low levels of parathyroid hormone (PTH) (1). In the USA, the estimate of its incidence is 37 per 100,000 people/year, and the most common etiology is postsurgical hypoparathyroidism, followed by autoimmune diseases and genetic disorders (2). In Europe, Danish studies have shown that the prevalence of postsurgical hypoparathyroidism is around 22/100,000 inhabitants (3) and that of nonsurgical hypoparathyroidism is around 2.3/100,000 inhabitants (4). All studies have shown a higher prevalence among women above 45 years old. Clinical features vary depending on the etiology and the rate of serum calcium decline. Acute hypocalcemia tends to lead to more exuberant manifestations whereas chronic reductions may go clinically unnoticed (5). Patients may present with neuromuscular, psychiatric, neurological, renal, cardiovascular, or ophthalmological symptoms, aside from the bone alterations (1).

Calcium plays a central role in many vital processes, such as muscle contraction, blood coagulation, neurotransmission, and hormone and enzyme activities, so its concentration is precisely regulated (6). When the parathyroid glands are compromised, PTH production and release are jeopardized, failing to rise in response to hypocalcemia. In this setting, little or no mobilization of the skeleton or increase in renal resorption occurs to increase calcium levels along with inadequate conversion of vitamin D to its active form, 1,25-dihydroxyvitamin D, which in turn increases the gut absorption of calcium (1). Also, PTH inhibits renal tubular reabsorption of phosphate (7–9), and its absence tends to enhance serum levels of phosphorus.

Replacement with the missing hormone is still not the first choice treatment for hypoparathyroidism. In most cases, patients take calcium and vitamin D supplements to maintain adequate serum levels of calcium and phosphorus while trying to keep calciuria within the normal range. In this review, we aim to discuss the currently recommended therapy and to present the novel and upcoming options to treat this rare condition. We conducted a literature search using the PubMed database and textbooks to identify articles, guidelines, and consensuses using the keywords “hypoparathyroidism”, “treatment”, and “management” as well as specific terms, such as “PTH analogs” and “calcilytics”.

## LONG-TERM TREATMENT GOALS AND MONITORING

Prompt identification and adequate management of hypocalcemia are important to prevent acute and long-term complications while controlling the patient’s symptoms. It is crucial to confirm hypocalcemia using either the corrected calcium levels for serum albumin, i.e., measured total calcium + [0.8 x(4-measured serum albumin)] or the ionized calcium concentrations. When measuring the latter, it is recommended that the sample be collected without prolonged restriction by the tourniquet (1). Also, PTH is relatively unstable, so adequate blood sampling and storage conditions are recommended (10). Clinical manifestations, laboratory data, and signs of chronic complications should be periodically assessed to reach the ideal balance for each patient, avoiding situations that can increase the risk of soft tissue calcifications (1,5). For many years, the conventional management method of hypoparathyroidism has been the use of calcium and vitamin D, but during the last decades, novel therapeutic options have emerged (Table 1).

**Table 1:** Treatment options for hypoparathyroidism

Drug	Dose
Calcium carbonate	2.5-7.5 g/day divided into 2-3 doses with meals
Calcium citrate	5-15 g/day divided into 2-3 doses; preferred in hypochloridria
Calcitriol	0.25-2 mcg/day divided into 2-3 doses
Cholecalciferol (with calcitriol)	400-4,000 UI/day
Cholecalciferol (alone)	10,000-100,000 UI/day
Hydroclothiazide, chlortalidone	12.5-100 mg/day in the morning
Magnesium oxide	250-1,000 mg/day divided into 2-4 doses
Sevelamer	800 mg three times a day with meals
PTH (1-84)	25-100 mcg/day

The treatment goal for patients with chronic hypoparathyroidism is to keep them asymptomatic and to maintain the calcemia slightly below or in the lower normal range and the calciuria up to 4 mg/kg/day (11) [if the patient is obese, consider the ideal body weight (7)] or 24-hour urinary calcium < 300 mg/24 h in men and < 250 mg/24 h in women (7,9,11–15). Serum phosphorus should be kept near the maximum value (11) but within the reference range (5,8,9,13,14,16).

If the patient is asymptomatic, a laboratory evaluation should be conducted every 6 months (7–9,11,12,15) and no less than every 12 months (5,14,17). If the dose of calcium and/or vitamin D supplements changes, serum calcium and phosphorus should be measured within 2 weeks (8,9). During pregnancy, serum phosphate should be evaluated every 2-3 weeks and every 4 weeks during lactation (8).

Doses of the active form of vitamin D should be carefully titrated, for it increases intestinal absorption of calcium through activation of the Ca2+-binding protein but also acts on the sodium-dependent phosphate co-transporter type IIb, enhancing phosphorus intestinal absorption and the risk for elevated calcium-phosphorus product. Therefore, either because of the disease itself or the high doses of calcium and vitamin D needed to control it, patients with chronic hypoparathyroidism are at risk for ectopic calcifications, often presenting with cataracts, nephrolithiasis, or calcifications in the basal ganglia. Kidney function should be evaluated periodically with estimated glomerular filtration rate and imaging (ultrasound, CT scans), and brain imaging should be requested if the patient presents with symptoms (5). Also, we should mind any opportunistic scans, such as thoracic or abdominal CT or DXA scans, which may show aortic calcifications, for example.

## CALCIUM SUPPLEMENTATION

The absence of the PTH effects on the gastrointestinal tract, kidney, and bones, leads to hypocalcemia and hyperphosphatemia. Oral calcium administration aims to increase serum calcium levels and to bind dietary phosphorus in the intestines, reducing its absorption. Therefore, ingestion of dairy products should be paced because although they are an excellent source of calcium, they are also rich in phosphorus (11).

Several types of calcium supplements are available, and the amount of elemental calcium and its biodisponibility vary among them. The most common forms are calcium carbonate (40% of elemental calcium) and calcium citrate (21% of elemental calcium). When reduced gastric acidity is present (e.g. gastrectomy, use of proton pump inhibitors) or when the patient complains of constipation with calcium carbonate, calcium citrate is preferred despite its lower elemental calcium concentration and need for more capsules per day (5). There are also calcium lactate and calcium gluconate (13% and 9% elemental calcium, respectively), but they are less used in clinical practice. Calcium phosphate should be avoided in patients with hypoparathyroidism (11).

Daily calcium intake recommendations vary by age, gender, and country, but the daily doses prescribed for patients with hypoparathyroidism depend on their calcemia and calciuria. Patients usually take 1 to 3 g of elemental calcium divided into 2-3 doses per day with meals, but it can go as high as 9 g per day (5).

In the setting of acute symptomatic or marked (<7.0 mg/dL) hypocalcemia, 1 to 2 g of calcium gluconate in 50 mL of 5% dextrose or saline can be slowly administered intravenously over 10 to 20 minutes. The acute administration only sustains the calcemia for some hours, and it should be followed by a calcium maintenance infusion of 0.5 to 1.5 mg/kg/hour over an 8-10-hour period, and the oral treatment should be optimized (1).

## VITAMIN D AND ITS ACTIVE METABOLITES

Vitamin D has 2 inert forms, cholecalciferol (or vitamin D3) and ergocalciferol (or vitamin D2), which can be acquired from diet; however, few foods, such as oil-rich fish, have them in adequate levels (18–20). They require a 2-step enzymatic hydroxylation to become biologically active. The first hydroxylation occurs in the liver, converting cholecalciferol to 25hydroxyvitamin D [25(OH)D], and the second one takes place in the kidneys, where 1α-hydroxylase converts 25(OH)D to 1,25(OH)_2_D (or calcitriol), the active form of vitamin D. Calcitriol production is strictly controlled; it is stimulated by PTH and hypocalcemia and inhibited by fibroblast growth factor-23 (FGF-23) and hyperphosphatemia (11,18–20).

PTH deficiency jeopardizes the activation of renal 1-α-hydroxylase, so the conventional treatment of hypoparathyroidism includes the use of calcitriol (5,11,21). It has a half-life of 4-6 hours, and the increase in serum calcium concentrations typically follows 1-3 days later (11,21). The usual dose is 0.25 to 2.0 mcg daily, which reflects the normal daily production rate of calcitriol, with larger doses rarely necessary. It should be divided into at least two daily doses and be titrated according to calcium levels (5,11,21,22). Alfacalcidol, a calcitriol analog, is rapidly activated in the liver to 1,25(OH)_2_D and has half the strength of calcitriol, with the same time to onset of action, of which the usual dosage ranges from 0.5-3.0 mcg daily (21).

Whereas the use of calcitriol is well accepted, the use of cholecalciferol or ergocalciferol is controversial. They were widely used at high doses (25,000- 200,000 IU daily) in the past, when the use of calcitriol was restricted (5,11,21). Currently, these forms of vitamin D can be prescribed with calcitriol given that many tissues generate their own 1,25-dihydroxyvitamin D and there are other metabolites of vitamin D, independent of PTH action, with supposed extraskeletal benefits, in addition to the fact that 25(OH)D occupies vitamin D receptors and mimics calcitriol activity, contributing to the maintenance of calcemia (1,5,21). Due to its longer half-life (2-3 weeks), the increase in serum calcium occurs more slowly (about 10 days), and in the case of intoxication, it can lead to prolonged hypercalcemia (5,7,11,21). Depending on the degree of vitamin D deficiency, a starting mega-dose of 50,000 IU weekly for 8 weeks may be necessary. The maintenance dose is usually 400-4,000 IU daily for patients using associated calcitriol, but special groups, such as obese or post-bariatric patients, may need doses up to 10 times higher (18,19,22). If calcitriol is not available, the daily dose of cholecalciferol is usually 10,000-100,000 IU (7,18,19,22).

Serum 25(OH)D is the most reliable indicator for monitoring vitamin D levels (18–20) and should be kept within the normal range, which is ≥ 20 ng/mL (50 nmol/L) (1,7,9) for the general population and ≥ 30 ng/mL (80 nmol/L) (21) for patients with bone diseases. In the setting of hypoparathyroidism, recommended cutoff values vary among studies (1,7,9,21). Levels up to 60 ng/mL are the target in patients using combined calcitriol, and levels > 80 ng/mL may be accepted in severely ill patients using cholecalciferol alone (7).

## THIAZIDE DIURETICS

In a healthy adult with normal renal function, about 60% of serum calcium is transported into the glomerular filtrate, and approximately 97.5% of it is reabsorbed (65% at the proximal renal tubule, 25% at the thick ascending limb of Henle’s loop, and 8% at the distal renal tubule) (16). PTH stimulates renal tubular reabsorption of calcium, as does calcitriol, and inhibits renal tubular reabsorption of phosphate (7–9). In hypoparathyroidism, besides the reduced production of calcitriol, PTH1Rs are not stimulated, leading to an upregulated action of claudin 14, which reduces claudins 16 and 19, resulting in decreased renal tubular resorption of calcium in the thick ascending limb of Henle’s loop (8,16). The resulting urinary calcium loss may aggravate hypocalcemia and increase the risk of nephrolithiasis, renal dysfunction, and nephrocalcinosis (7–9,11,17).

The dose-dependent natriuretic action of thiazide diuretics in the distal renal tubule leads to a compensatory mechanism that results in increased passive calcium transfer, increasing natriuresis and enhancing renal calcium resorption by approximately 6% (9,12,14–16). Therefore, this drug class can be an ally in the treatment of hypercalciuria in patients with hypoparathyroidism (5,7–9). The most commonly prescribed drugs to treat hypercalciuria in patients with hypoparathyroidism are hydrochlorothiazide, chlorthalidone (17), and indapamide (8). The recommended starting doses for hydrochlorothiazide and chlorthalidone are 12.5 mg once daily (15), 25 mg once daily and 12.5 mg twice daily (11), with a maximum dose of 50 mg twice daily (9,11).

Side effects should be monitored, such as hypokalemia, hypomagnesemia (5), hypercalcemia, hypotension, hyponatremia (9), increased urinary sodium loss (7,12–14,17), and elevation of serum uric acid (15). Thiazide diuretics are contraindicated in patients with adrenal insufficiency (5,13) and patients with autosomal dominant hypocalcemia (activation mutation in the calcium-sensing receptor) who also present with Bartter’s syndrome (5). Thiazide diuretics should be avoided during pregnancy (8).

A low-sodium diet should also be recommended. Sodium transporters in the proximal renal tubule resorb calcium from the glomerular filtrate along with sodium, independently of PTH. Therefore, a low-sodium diet reduces natriuresis and leads to renal reabsorption of sodium and calcium (5,7–9,13,14,16).

In patients with hypoparathyroidism, loop diuretics, such as furosemide, should be used with caution, as they act on the thick ascending limb of Henle’s loop, simultaneously decreasing sodium and calcium reabsorption and increasing urinary calcium loss (11,12,16,17). If clinically possible, it should be discontinued (12,15,17). This discontinuance also applies to glucocorticoids, as they induce hypercalciuria and impair the action of vitamin D and its analogs (11,12).

## PHOSPHORUS BINDERS

In a healthy person with normal renal function, about 95% of serum phosphorus is transported into the glomerular filtrate, and the proximal renal tubule reabsorbs approximately 80% of the phosphorous through the action of sodium-phosphate transporters, mainly NaP2a and NaP2c. In hypoparathyroidism, the lack of PTH action increases the activity of NaP2a and NaP2c, increasing renal resorption of phosphate. Although hyperphosphatemia increases fibroblast growth factor 23 (FGF23, a hormone that inhibits the action of NaP2a and NaP2c, decreasing renal tubular resorption of phosphorus), this increase is not enough to compensate for the lack of PTH (16).

To decrease intestinal absorption of phosphorus, a low-phosphorus diet is recommended (5,9,11,12,14,17), avoiding cola drinks, sausages, meats, and eggs (8,12,13). Dairy products should be consumed with caution (7,8,11–13).

Dietary measures and calcium intake at meals are usually enough to control the phosphatemia, as well as intestinal phosphorus binder drugs that do not contain calcium (5,11,13,17), such as sevelamer and lanthanum carbonate (7), which are only needed in the case of refractory hyperphosphatemia. Sevelamer is an allylamine hydrochloride polymer, which binds to phosphorus in the intestinal lumen. The starting dose is 800 mg three times a day with meals, and the possible side effects are constipation, dyspepsia, nausea, abdominal pain, diarrhea, and flatulence (7,11,12,14,17).

## MAGNESIUM

Hypermagnesemia can decrease PTH action (5,15) through the adenylcyclase system and suppress PTH secretion by stimulating calcium-sensing receptors (8,9,11). Interestingly, hypomagnesemia can also block PTH synthesis and secretion by increasing the activity of the inhibitory G-alpha subunit (8). Hypermagnesemia is rare, being mainly associated with renal failure (8,15), and so far, the only treatment besides dialysis is to restrict the dietary intake by avoiding magnesium-rich foods, such as legumes and whole grains. In case of hypomagnesemia, magnesium can be replenished with oral formulas containing magnesium salts (9,11).

The recommended magnesium oxide dose (60% elemental magnesium) is 250-1,000 mg divided into 2 to 4 daily doses (7). The daily dose of magnesium pidolate (130 mg of elemental magnesium) is 1 to 2 capsules or vials per day (11). Some possible side effects of magnesium replacement are hypermagnesemia, nausea, diarrhea, and gastritis (9,17). Amiloride is a potassium-sparing diuretic that decreases urinary magnesium loss and can be prescribed when intolerance to oral magnesium supplementation occurs (9,17).

The lack of PTH effect itself may contribute to hypomagnesemia because PTH increases the resorption of magnesium in the distal renal tubule (7,17), but other causes of chronic hypomagnesemia should always be investigated. Drugs, such as proton-pump inhibitors, thiazide diuretics, and loop diuretics (8,9), and clinical situations, such as chronic diarrhea, alcoholism, gastrointestinal malabsorption (11), low-magnesium diet (8), and increased renal loss of magnesium (as in Fanconi syndrome, renal tubular necrosis, and poorly controlled diabetes mellitus) (15), may also decrease serum magnesium levels and should be dealt with.

## PTH ANALOGS

Hypoparathyroidism persists as the last hormone deficiency in which conventional treatment is not performed with the use of the missing hormone (23). The first studies with PTH analogs were conducted by Winer and cols., who treated adults with PTH (1–34), initially with a single daily dose (24) but switched to two daily doses due to its short half-life (25,26). In 3-year studies, researchers compared it to conventional treatment in children and adults (5-70 years), reporting more stable maintenance of calcemia and magnesemia with no differences in urinary calcium (23,27).

A phase III, open-label, non-comparative study showed that teriparatide 20 µg once daily was insufficient to discontinue calcium and calcitriol supplements to maintain normal calcemia. On the other hand, when using teriparatide 20 µg twice daily, more than half of the patients were able to stop taking calcium and calcitriol (28).

The use of PTH (1–34) seems to stimulate bone turnover, but reduced BMD of the distal radius has been reported (29), and no long-term safety data are available. It is important to emphasize that PTH (1–34) is approved for the treatment of osteoporosis, but its use in hypoparathyroidism is off label (30). Warnings about the risk of osteosarcoma still remain, especially in children, but so far, there is no solid evidence of its occurrence in humans.

In January 2015, the FDA approved PTH (1-84) for the treatment of hypoparathyroidism in patients with inadequate control with conventional treatment. From a pharmacokinetic point of view, this molecule has been shown to have a longer half-life, allowing for a single daily application. Studies have shown that a dose titration of 50, 75, and 100 mcg is necessary, thus enabling the gradual reduction in calcium and vitamin D doses. Monitoring of calcemia is necessary, as episodes of hypercalcemia have been described, mostly asymptomatic, especially with the 100-mcg dose. In some cases, complete discontinuation of calcium and vitamin D supplements was possible (31–33). The study with the longest duration was that of Tay and cols., with 24 patients followed for 8 years. Calciuria decreased (38%), calcemia, and phosphatemia were maintained, and BMD increased at the lumbar spine and total femur sites but with a 33% decrease in the radius BMD. The calcium and calcitriol doses decreased by 57% and 76%, respectively, with 50% of the subjects able to discontinue the use of calcitriol (34). The REPLACE study is a double-blind, randomized, placebo-controlled, phase 3 study that reported the proportion of adult patients (18-85 years) who were able to reduce their calcium and vitamin D doses by 50% after 24 weeks - 53% in the PTH group (1-84) versus 2% in the placebo group (35).

The use of PTH analogs in the treatment of hypoparathyroidism is promising, but it is a high-cost injectable therapy that is currently indicated for patients in special situations, such as those using high doses of calcium and vitamin D without proper control, with poor adherence, or with the presence of gastrointestinal disorder with malabsorption. The studies showed no complete suspension of calcium and vitamin D in most patients but showed dose reduction. No data are available regarding the use of PTH (1-84) in children. More studies are needed to assess long-term neurocognitive outcomes, fractures, nephrocalcinosis, and nephrolithiasis events (26,36).

## PREGNANCY

It is critically important to avoid fluctuation in maternal serum calcium levels during pregnancy due to potential adverse events for the mother and the fetus. Fetal hypocalcemia stimulates the fetal parathyroid glands, causing compensatory hyperparathyroidism and subsequent demineralization of the fetal skeleton with intrauterine rib and limb fractures. Low birth weight and intrauterine fetal death have also been described in women with hypocalcemia during pregnancy. On the other hand, maternal hypercalcemia can lead to suppression of the fetal parathyroid glands, resulting in fetal hypocalcemia, which may be associated with neonatal seizures. Thiazide diuretics and PTH analogs, both (1–34) and (1-84), should not be used during pregnancy. Elemental calcium, active vitamin D, and vitamin D supplements are safe to use during pregnancy (37).

## PERSPECTIVES

TransCon PTH is a once-daily long-acting prodrug of PTH for adult hypoparathyroidism treatment. The prodrug consists of a PTH (1–34) molecule, transiently bound to an inert carrier via a proprietary linker. The bonding hinders the binding of the parent drug to the receptor and its uptake as its renal clearance and enzymatic degradation. Following the injection and on exposure to physiologic conditions, autocleavage of the linker occurs, and active PTH is released in a controlled way. The placebo-controlled phase 2 trial showed that TransCon PTH enabled independence from oral active vitamin D and reduced Ca supplements (≤500 mg/day) for most participants (91%), achieving normal serum calcium, serum phosphate, urinary calcium, and serum calcium-phosphate product and demonstrating improved health-related quality of life (38).

Calcilytics are negative allosteric modulators of the extracellular calcium receptor (CaR). The NPSP795 molecule (encaleret) increased plasma PTH levels in a concentration-dependent manner up to 129% above baseline in 5 patients with autosomal dominant hypocalcemia type 1 (ADH1). NPSP795 was generally safe and well-tolerated (39).

Finally, allotransplantation, stem cell therapy, and microencapsulation of human parathyroid cells are experimental techniques under investigation (40).

In conclusion, hypoparathyroidism is a rare disease with limited treatment options. Conventional treatment involves the use of calcium salts and vitamin D analogs. Calcium carbonate is the most available form and needs to be administered with meals to increase its absorption whereas calcium citrate does not have the same requirement. Calcitriol is administered in doses ranging from 0.5 to 2.0 mcg per day. Vitamin D deficiency should be corrected with vitamin D3. Thiazide diuretics can reduce hypercalciuria in some cases. A low-phosphate diet is recommended, and phosphate binders are rarely needed. During pregnancy, a careful approach is necessary to avoid hypo- and hypercalcemia, may have deleterious effects on the fetus. A new approach to the treatment of hypoparathyroidism is the use of PTH analogs limitations imposed by the high cost.
